# Factors Influencing Parent and Guardian Decisions on Vaccinating Their Children Against SARS-CoV-2: A Qualitative Study

**DOI:** 10.1177/00469580231159742

**Published:** 2023-03-20

**Authors:** Andrea Nickerson, Luis Gutierrez-Mock, Laura Buback, Susie Welty, Lynhea M. Anicete, Sabrina Sanchez, Wayne T.A. Enanoria, Mike Reid

**Affiliations:** 1 University of California, San Francisco, CA, USA; 2 San Francisco Department of Public Health, San Francisco, CA, USA

**Keywords:** COVID-19 vaccination, childhood vaccines, health communication, vaccine hesitancy, vaccine campaigns

## Abstract

This qualitative analysis sought to explore factors that influenced parent/guardian intentions to vaccinate their children against SARS-CoV-2 in San Francisco, California, USA in order to inform San Francisco Department of Public Health’s (SFDPH) youth vaccine rollout program. 30-minute, semi-structured telephone interviews were conducted with parents and guardians in either Spanish or English. Respondents shared their perspectives on vaccinating their children against SARS-CoV-2. Interviews were conducted over the telephone and recorded on Zoom. Participants (n = 40) were parents/guardians responding on behalf of their adolescent children (age 13+) and parents/guardians identified from the SFDPH COVID-19 testing database who tested for SARS-CoV-2 within the last 2 weeks. Interviews were conducted, audio recorded, transcribed, translated into English as appropriate, and rapidly analyzed in REDCap according to matrix analysis methodology to develop parent study themes. Perspectives on child vaccination were then explored through thematic analysis. Three themes were identified from the thematic analysis: (1) parental desires for children to return to school safely, (2) unclear messaging and information on COVID-19 prevention and vaccination, and (3) consideration of child’s desires or opinions on receiving the vaccine. This study highlights specific factors influencing parent/guardian decisions on whether to vaccinate their children against SARS-CoV-2. The analysis also illustrates a potential role for children to play in influencing household vaccine decision-making.

**What do we already know about this topic?**
Vaccinating children against SARS-CoV-2 has psychosocial and societal benefits, but widespread vaccine hesitancy among parents and guardians is impeding vaccine rollout.
**How does your research contribute to the field?**
This focused analysis adds to a limited body of qualitative research exploring factors influencing vaccine uptake among school-aged children in the US; it highlights real-world perspectives of parents/guardians and the importance of children’s opinions on COVID-19 vaccine uptake.
**What are your research’s implications toward theory, practice, or policy?**
The results of this illuminate the impact of clear, consistent public health communication, targeted to both parents and their children, on vaccine uptake in children.

## Purpose

Vaccines are critical tools to achieving epidemic control of SARS-CoV-2, the virus responsible for the COVID-19 pandemic. Of the available vaccines in the United States, the Pfizer-BioNTech vaccine was approved for emergency use in adolescents aged 12 to 15 years in May 2021,^1^ in children aged 5 to 11 years in late October 2021,^2^ then subsequently infants and children aged 6 months to 4 years in June 2022.^3,4^ Furthermore, by December 2022 bivalent boosters were approved for all ages, including children down to 6 months of age.^5^ Nevertheless, in many parts of the US, vaccine coverage has been undermined by widespread vaccine hesitancy.^6,7^ As of December 15th, 2022, 69% of eligible people have completed the primary vaccine series and only 14% have updated bivalent booster doses.^8^

Increasing overall vaccine coverage is greatly needed, particularly as new variants continue to emerge.^9,10^ There is an urgent need to increase coverage in children specifically, given the protective benefits and the broader epidemiologic dividend of vaccinating children.^11^ Unfortunately, in a recent survey of 1847 US adults, only 62% thought COVID-19 vaccines were safe for children ages 12 to 17, 55% thought they were safe for children ages 5 to 11, and 48% thought they were safe in very young children (0-4).^12,13^ In San Francisco, 86% of residents of all ages and at least 90% of 12-to-17 year-olds completed the initial series against SARS-CoV-2.^14^ However, vaccine coverage in children ages 5 to 11 lags at 79% and 24% for children 0-4.^15^ Because children in most states need parental consent to get vaccinated,^16^ it is critical to understand parent/guardian concerns to better inform targeted vaccine promotion efforts.

This focused analysis explores the parent/guardian decision making process with regards to vaccination of their children in San Francisco, California. Understanding these perspectives can help inform strategies to maximize vaccine coverage, including booster uptake, among children. While this study was conducted in the summer of 2021, a time when vaccines were not yet approved for children under 12 years old, the findings remain pertinent as vaccines and boosters continue to be one of the most important defenses against existing and emerging pathogens.

## Methods

### Design and Setting

The data for this paper were obtained from a mixed-methods study on barriers and facilitators to COVID-19 testing and COVID-19 vaccine hesitancy in San Francisco. All participants were San Francisco residents over the age of 13. Participants were guided through a survey and a semi-structured qualitative interview by trained study staff over the phone, which were recorded on Zoom. This focused analysis explores a subset of participants that are parents of children under 18 years old and/or guardians of minor children.

### Participants

The sampling frame for this study included all people who tested for COVID-19 within the San Francisco Department of Public Health’s (SFDPH) COVID-19 testing database from July to August 2021. Name, date of birth, sex, telephone number and test results were extracted weekly from the SFDPH database and securely transferred to the study team’s systems. Indeterminate test results were excluded, and participants were randomized in the sampling frame. The study team called potential participants within 2 weeks of their test dates and discussed study goals, assessed eligibility and gained verbal informed consent for participation and recording of interviews. Participants were contacted within 2 weeks of their COVID-19 test date in order to reduce recall bias in the quantitative portion of the larger mixed-methods study^17,18^ and to ensure that SFDPH COVID-19 case investigators were able to contact positive cases with isolation guidance.

Eligibility criteria included: being a San Francisco resident; ability to speak English, Spanish, Mandarin, Tagalog, Hindi or Telugu; ability to provide date of birth and COVID-19 test result that matched the testing database information; self-reported test result was either negative or positive; participants with a positive test result reported being notified about their result and provided isolation instructions (positive individuals who had not received their test result or isolation guidance were ineligible and provided a warm handoff to the San Francisco COVID-19 case investigation team); and the participant or their parent/guardian was over the age of 18. Individuals in the sampling frame between the ages of 13 and 17 were included, however, our protocol and IRB approval required that the study team speak with their parent or legal guardian over the age of 18. The parent/guardian then responded on behalf of the minor. Parents/guardians were probed about their decision-making processes to vaccinate or not vaccinate their children.

For this focused analysis, data from individuals who did not report having children under 18 (n = 43) were excluded. All participants were asked if they had children under the age of 18. Any participants that answered “Yes” to having children of any age under the age of 18 and any parents/guardians responding on behalf of their 13- to 17-year-old adolescent child were included in this analysis. A total of 40 participants met these criteria.

### Data Collection

Eligible participants completed a 30- to 35-minute, survey and semi-structured telephone interview conducted by a member of the study team between July 20 and August 31, 2021. Participants with children under the age of 18, including parents/guardians responding for their minor children in our sampling frame, were asked to discuss their perspectives on vaccinating their children against COVID-19, including if their eligible children were vaccinated, why or why not, any barriers or hesitancies they faced, and their perspectives on specific San Francisco vaccine mandates. At the time of our study, only children 12 and older were eligible for vaccination, but parents/guardians were probed for perspectives regarding potential upcoming vaccinations for any children they had under 12 years old. Responses were entered directly into a secure REDCap electronic data form and audio recorded. Interviews were conducted in English or Spanish (although there was capacity to provide interviews in additional languages, no participants requested interviews in those languages), and participants were compensated for their time with a $40 gift card following the completion of the interview.

### Data Analysis

Interview recordings were transcribed, and all identifying/personal health information redacted. Spanish interviews were transcribed into Spanish text, analyzed in Spanish and then translated into English. Following transcription, the study team used rapid matrix analysis methodology,^19,20^ to analyze key topics based on similarities, differences and inter-relatedness to identify initial themes, and reported these back to SFDPH 17 days after data collection ended. Four senior study staff members conducted the rapid matrix analysis, utilizing a summary template in REDCap (Supplemental File 1) to summarize and analyze transcripts. These 4 staff members completed several summary templates together, then individually completed summary templates for the same participants until summary alignment was met. The summary template included demographics, reason(s) for testing, and a summary of parent/guardian perspectives on getting their children vaccinated against COVID-19 and how public health measures had impacted their children. Rapid matrix analysis findings were triangulated through an intensive team-based analysis among the 4 senior study members during and immediately after data collection. We additionally met regularly to discuss our analysis with the 8 research assistants conducting interviews. One key topic generated from the rapid matrix analysis was parental vaccine deliberation for minor children, which is further explored in this focused analysis.

Building from the rapid matrix analysis findings, thematic analysis^21^ was used to focus on parental vaccine deliberation for minor children. The lead author analyzed and coded all English transcripts for content related to parent/guardian vaccine deliberation in minors in Dedoose. A Spanish-speaking study team member analyzed and coded the Spanish transcripts in Spanish and checked English language translations for accuracy. The lead author then wrote memos and summary sheets for each of the 40 included participants regarding parental vaccine deliberation. Overall summaries and preliminary themes were discussed with the analysis team. The analysis team triangulated the final themes through iterative team-based discussions, that looked at the frequency of theme occurrence and relevance to public health policy in San Francisco per our discussions with SFDPH regarding our rapid matrix analysis findings.

### Ethical Considerations

Written consent was waived by IRB and verbal informed consent to participate was gained from all participants. This study was approved by the lead author’s IRB, #21-34529.

## Results

A total of 40 interviews with perspectives on vaccinating minor children were analyzed. Parents/guardians responded for their adolescent (age 13-17) participants in the sample frame (n = 28), and 12 other parent/guardian participants within the sample frame gave perspectives about vaccinating their non-participant minor children. Vaccination information was collected for all participants in the sample frame, as well as any additional children that the parents/guardians had that were outside of sample frame. Demographic and vaccination information for the study participants and minor children represented in these interviews is summarized in Tables 1 and 2.

**Table 1.** Demographic Information for Study Participants.

	Youth in sample frame (n = 28)	Parents in sample frame (n = 12)
Mean age in years (SD)	14.8 (1.5)	42.5 (6.7)
Race/ethnicity (%)		
AI/AN	0 (0)	1 (8.3)
Asian	1 (3.6)	0 (0)
Black or African American	6 (21.4)	1 (8.3)
Native Hawaiian	1 (3.6)	0 (0)
White	9 (32.1)	7 (58.3)
Multiracial	4 (14.3)	2 (16.7)
Other	6 (21.4)	1 (8.3)
Do not know	1 (3.6)	0 (0)
Occupation*,+ (%)		
Construction	1 (14)	0 (0)
Food service	0 (0)	3 (25)
Government/community	0 (0)	1 (8.3)
Healthcare, emergency services	1 (14)	3 (25)
Manufacturing	0 (0)	1 (8.3)
School childcare education	0 (0)	1 (8.3)
Transportation logistics	0 (0)	1 (8.3)
Other	2 (28)	0 (0)
Unemployed	1 (14)	2 (16.7)
Retired	2 (28)	0 (0)
Gender (%)		
Female	11 (39.3)	5 (41.7)
Male	15 (53.6)	6 (50)
Genderqueer or non-binary	2 (7.1)	0 (0)
Trans male, trans man	0 (0)	1 (8.3)
Sexuality** (%)		
Heterosexual or straight	–	11 (91.7)
Bisexual	–	1 (8.3)

* Occupation is that of the parent/guardian respondent.

+ Only 7 parent/guardian respondents were asked about their occupation.

** Parent/guardian respondents were not asked about their children’s sexual orientation.

**Table 2.** Vaccination Status of Study Participants and Additional Children.

	Age	Vaccinated (%)	Unvaccinated (%)	Ineligible (%)
Minors in sample frame (n = 28)	13-17	18 (64.3)	10 (35.7)	–
Minors outside of sample frame (n = 43)	<12	–	–	26 (100)
	13-17	14 (82.4)	3 (17.6)	–
Parents in sample frame (n = 12)	18+	10 (83.3)	2 (16.7)	–
Total	–	42 (50.6)	15 (18.1)	26 (31.3)

Three themes related to factors influencing parent/guardian COVID-19 vaccine deliberation for their children were identified: (1) Parental desires for children to return to school safely, (2) Unclear messaging and information on COVID-19 prevention and vaccination, and (3) Consideration of child’s desires or opinions on receiving the vaccine.

### Parental Desires for Children to Return to School Safely

Returning to school was a frequent topic of discussion amongst the parents/guardians interviewed. It was important for many parents because their children had “lost a year of normal childhood experiences” already, and many children suffered detrimental mental health outcomes due to the severe disruption to their social and learning routines. Parents described simultaneously wanting their children to return to school but also worrying about the increased COVID-19-risk related to being back in school:

*“I don’t know. . . it’s very risky. I mean you want. . . your kids to go to school, get an education, you know, not fall behind, but at the same time, it’s like very dangerous and risky. It’s like a catch 22 in a way. Damned if you do, damned if you don’t. If your kid’s out of school, they fall behind in education, you put them in school, they take, you know, their health could be at risk.” – Parent of a 14-year-old and under 10-year-old children*

Parents of children eligible for vaccination at the time of interview cited protecting their children from the increased COVID-19 risk as they returned to school as one reason their children were vaccinated.

Returning to school also provided a motivation for initially hesitant parents to vaccinate their children. Some parents reported being initially hesitant to get their child vaccinated but explained their reasons for changing their minds, which again included a desire to protect their children from the perceived risk of returning to highly social situations or because of the expectation that vaccines would be mandated among school-aged children:

*“When I had to [get my child vaccinated] for school, I finally came around, I thought, you know, it’s better for him to be safer because he’s going to be in high school, and he’s going to take the bus lots of places. So, we decided to go ahead and do that.” – Parent of a 14-year-old*

Parents of younger children (<12 years old), ineligible at the time of the study, who were eagerly awaiting vaccine recommendations for their children were pleased that vaccines were mandated for teachers and school employees. This made them feel that their children were still somewhat protected, as one mother highlighted.

*“I’m glad that the school district is [requiring vaccination]. It makes me feel more confident in, like, having my kids in school. So, I do feel really good about the school district requiring it. . . I do actually really appreciate it. . . I mean, especially since they’re not vaccinated, of course.” – Parent of two children under 11-years-old*

### Unclear Messaging and Information on COVID-19 Prevention and Vaccination

Parents/guardians mentioned that unclear messaging with respect to COVID-19 policies in school and confusion about real-world vaccine effectiveness added to their fears and hesitancy about sending their children back to school and, ultimately, having their children vaccinated. Parents/guardians expressed concern over sending their children back to schools where specific COVID-19 risk reduction policies (masking, ventilation, exposure, etc.) were not clearly communicated. One mother describes her specific worries:

*“[The high school] was built in the, like, in the seventies. And there’s like a bunch of classes that don’t have any windows. And we don’t really know what the ventilation is like. I mean, they had to pass a ventilation certificate, but does that mean like HEPA filter? We have no idea. And we don’t know what the plan is for-Are they going to have masks outside? And what happens when people are sick?” – Parent of two 14-year-old children*

A specific COVID-19 related fear—the uncertainty of sick children being sent to school with or without knowing they had COVID-19—was mentioned by multiple parents. Through mostly anecdotal evidence, parents expressed distrust in the school system to effectively keep sick children or staff out of school; some parents stated that they had not received clear information describing plans to handle COVID-19 exposures at school. One participant, in addition to citing unclear information from schools, suggested that a lack of social support for some parents to take time off work to care for children suffering from COVID-19 may be exacerbating this specific issue.

Unclear messaging about prevention from schools, coupled with confusion about children still being susceptible to COVID-19 even after vaccination led parents to question the need for vaccination. This idea often came up in relation to the vaccination mandate for schools, leaving some parents feeling negatively about vaccines requirements. Together these gaps made parents doubt the overall benefit of the vaccine as one mother describes below:

*“I was talking to somebody yesterday, and she said that a little boy came to the school, and he got on the bus with COVID. So, if he took that vaccine just to get in school and he caught COVID, it’s like, what are you taking it for?” – Parent of a 2-year-old and 17-year-old*

Further, unclear information about the vaccines themselves led hesitant parents to feel confused, fearful, and ultimately reluctant to have their child vaccinated. Many hesitant parents wanted more information on long-term effects of the vaccine on children. A small number of parent/guardian participants (n = 7) cited the lack of information on long-term effects as a reason for being opposed to vaccinating their children. These parents were more likely to be unvaccinated against COVID-19 themselves. One parent describes his concerns about vaccinating himself and his children.

*“I’m* *not against vaccines – I’m* *not an early adopter. . .* *I’d just rather see how this washes out a bit. [Vaccinating my children under 12] will never happen.* *There’s 0% of that chance, there’s* *0% of that chance of happening. I would homeschool my children before I made them get this vaccine.* *There’s zero long-term studies on how it affects the developing body.” –Parent of two children under 12-years-old*

Without long-term studies, parents of younger children were uncertain about vaccinating their children regardless of school or other mandates.

Several parents expressed confusion with regards to the multiple vaccine options and differing vaccine eligibility for children of different age groups. One mother was already overwhelmed by the amount of information she needed to process to understand which of the 3 adult options was safest for her. Despite the confusion she felt surrounding her personal decision to get vaccinated, she also had to make further decisions for her children based on unclear child eligibility rules:

*“I would consider it for my youngest child if I get it, but right now they keep saying that 10-year-olds, under 12, can’t get the vaccination. Its raising more of awareness or concern rather. Why not, if the 12-year-old can have it? So just another kind of question that I’m worried about. They’re only two years apart. What’s the situation? What is the fear?” – Parent to a 10-year-old, 12-year-old, and 17-year-old*

In some cases, the fears and concerns that parents or children had were alleviated by speaking with trusted resources such as doctors or family members who were able to provide clear information and reassurance about vaccination.

### Consideration of Child’s Desires or Opinions on Receiving the Vaccine

**Figure 1.**
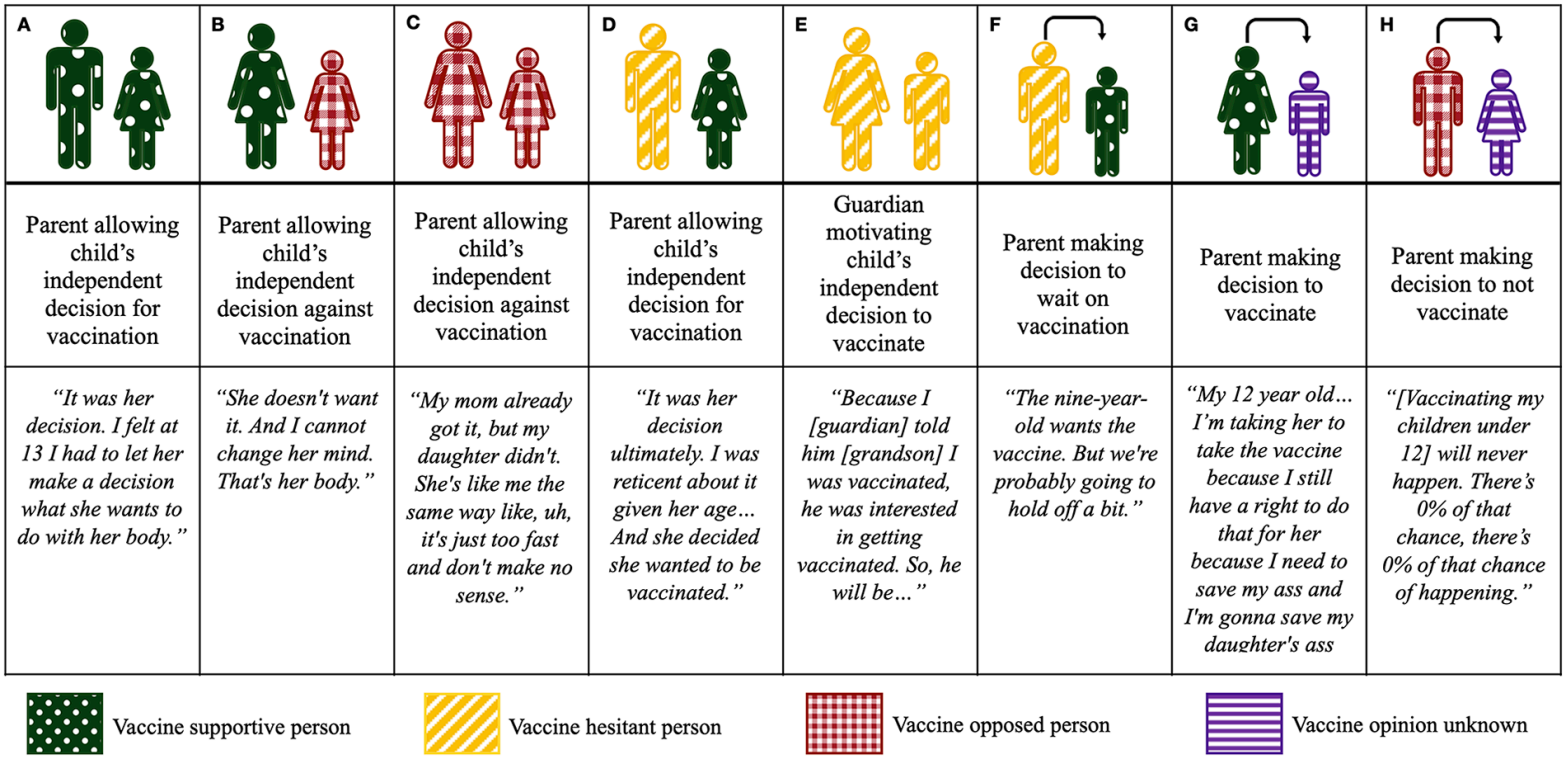
Vaccine decision making dynamics among study participants.

Another key theme concerned the role of adolescents and children in the vaccination decision making process. Most notably, parent/guardian and child views on vaccines were not always in agreement, and some parents/guardians let their children decide whether or not to be vaccinated, regardless of the parents’/guardians’ opinions and vaccination status. A summary of the decision dynamics among study respondents is outlined in Figure 1.

Most parents/guardians stated they made vaccination decisions on behalf of their children, without explicitly engaging their children in the decision-making process (Figure 1, categories G and H). However, a subset of parents reported that they would allow their child to make an independent decision to be vaccinated, even if that decision contrasted with the parent’s beliefs (Figure 1, categories A-E). This dynamic lead to outcomes of the child choosing to be vaccinated or choosing to remain unvaccinated. In one interview, the parent decided against the child’s desire to vaccinate (F), while in another, the guardian and grandchild motivated each other to overcome their own hesitancies (E).

## Discussion

In this qualitative analysis of factors influencing the parent/guardian decision making process with regards to vaccination of their children against SARS-CoV-2 among San Francisco-based parents and guardians, we found 3 factors that most often influenced that parents/guardians in our sample: parents’ intention to have their children safely return to school and social routines was an important motivator for vaccination; unclear, overwhelming, or incomplete public health messaging about COVID-19 policies and vaccines left many parents feeling concerned about the safety, effectiveness, and overall benefit of the vaccine, which lead to hesitation around vaccination; and the opinions of the children of the parents/guardians interviewed in this study often contributed, both for and against, to the decision to get vaccinated. Outlined below are the key findings and their policy implications.

Firstly, our analysis showed that getting children back into school was a key motivator for parents when considering COVID-19 vaccinations, often linked to improving their children’s mental health. Disruption of normal social life due to the pandemic and shelter-in-place orders in San Francisco, including school closures and transition to remote learning, exacerbated mental health issues for many of the study participants’ children, a finding supported by a decline in mental health in school-aged children^22^ and increased mental health-related hospital visits in children across the US during the pandemic.^22,23^ In this context, the prospect of getting their children vaccinated helped parents feel that their children would be better protected while in congregated, social situations, like schools, where the risk of SARS-CoV-2 spread and infection is higher.^24,25^ Additionally, the prospect of vaccine mandates at schools did help motivate some hesitant parents to have their children vaccinated.

Secondly, our analysis highlights how lack of clear information on COVID-19 prevention policies and vaccines remains a major barrier to parents willingly getting either themselves or their children vaccinated, even in settings, like San Francisco, with high vaccine coverage.^26^ These findings underscore the continued need for clear and consistent communication,^27^ including messaging specifically targeting parents of school-aged children. The participants in our study were highly invested in protecting their children but were often too confused about vaccine information to make a decision about vaccination. Lack of information from schools coupled with questions on the real-world effectiveness of vaccines, left parents feeling that the risk of vaccination was not worth the benefit. The continued relevance of these findings cannot be understated; with newer more, transmissible variants^28,29^ as well as development of bivalent vaccines,^30^ effective risk communication for parents, caregivers and those engaged in health promotion in schools remains highly relevant. Moreover, clear steps on how schools are planning to reduce COVID-19 risks, along with up-to-date safety and effectiveness data on vaccines, may help convince parents who do not want to vaccinate their children.^31,32^

Finally, our analysis highlights the interesting role children have in the vaccination decision making process. While some parents/guardians made the choice to vaccinate their children, our results show that a number of parents/guardians considered their children’s perspectives and supported their child’s independent decisions. These findings suggest youth-targeted vaccination campaigns and messaging could help children make informed decisions on COVID-19 vaccinations and advocate their desires to their parents. Promoting children’s role in the decision to vaccinate, in addition to clearer messaging, could be especially critical in overcoming parents’ hesitation to vaccinate their children leading to higher vaccine uptake.^33^

### Limitations

Our study is not without limitations. Given the small sample size, we cannot generalize these findings to settings beyond San Francisco. While select demographic questions were asked, the sample is also too small to stratify conclusions based on demographic differences in opinions or behaviors, in particular, based on race, social economic status, and ethnicity. Additionally, the unique context of San Francisco, with aggressive response strategies, a relatively high vaccination rate and higher acceptance of COVID-19 mandates must be considered when interpreting findings for other geographies. Our findings are also susceptible to selection bias, as we only included people who sought testing, responded to the phone call, and accepted participation in the study. This is a limited segment of the population and those excluded may have different opinions.

## Conclusions

This study adds to a limited body of qualitative research exploring factors influencing vaccine uptake among children in the United States by specifically exploring factors that influence the decisions of parents and guardians. Our findings are relevant given the expansion of age eligibility of children to receive COVID-19 vaccines, the release of bivalent or updated booster vaccines, as well as continued importance of new vaccines as a public health intervention.

Additionally, parents/guardians will continue to be decision makers for childhood vaccinations through and beyond COVID-19. The results underscore the need for clear vaccine promotion efforts targeting adolescents that highlight not only the protective benefits of the vaccine, but also prioritize how increased vaccine coverage may help improve psychosocial wellbeing for many children by allowing them to remain in routines and social networks. Finally, the results highlight the important role children can play as decision influencers and potential advocates for vaccination. As such, vaccine promotion efforts should engage children in addition to their parents.

## Supplemental Material

sj-docx-1-inq-10.1177_00469580231159742 – Supplemental material for Factors Influencing Parent and Guardian Decisions on Vaccinating Their Children Against SARS-CoV-2: A Qualitative Study Click here for additional data file. Supplemental material, sj-docx-1-inq-10.1177_00469580231159742 for Factors Influencing Parent and Guardian Decisions on Vaccinating Their Children Against SARS-CoV-2: A Qualitative Study by Andrea Nickerson, Luis Gutierrez-Mock, Laura Buback, Susie Welty, Lynhea M. Anicete, Sabrina Sanchez, Wayne T.A. Enanoria and Mike Reid in INQUIRY: The Journal of Health Care Organization, Provision, and Financing

sj-docx-2-inq-10.1177_00469580231159742 – Supplemental material for Factors Influencing Parent and Guardian Decisions on Vaccinating Their Children Against SARS-CoV-2: A Qualitative Study Click here for additional data file. Supplemental material, sj-docx-2-inq-10.1177_00469580231159742 for Factors Influencing Parent and Guardian Decisions on Vaccinating Their Children Against SARS-CoV-2: A Qualitative Study by Andrea Nickerson, Luis Gutierrez-Mock, Laura Buback, Susie Welty, Lynhea M. Anicete, Sabrina Sanchez, Wayne T.A. Enanoria and Mike Reid in INQUIRY: The Journal of Health Care Organization, Provision, and Financing
