# Supplementary figures and images for: Factors Influencing Parent and Guardian Decisions on Vaccinating Their Children Against SARS-CoV-2: A Qualitative Study
Source: Inquiry. 2023 Mar 20;60:00469580231159742. doi: 10.1177/00469580231159742 (PMC10031620; doi:10.1177/00469580231159742)

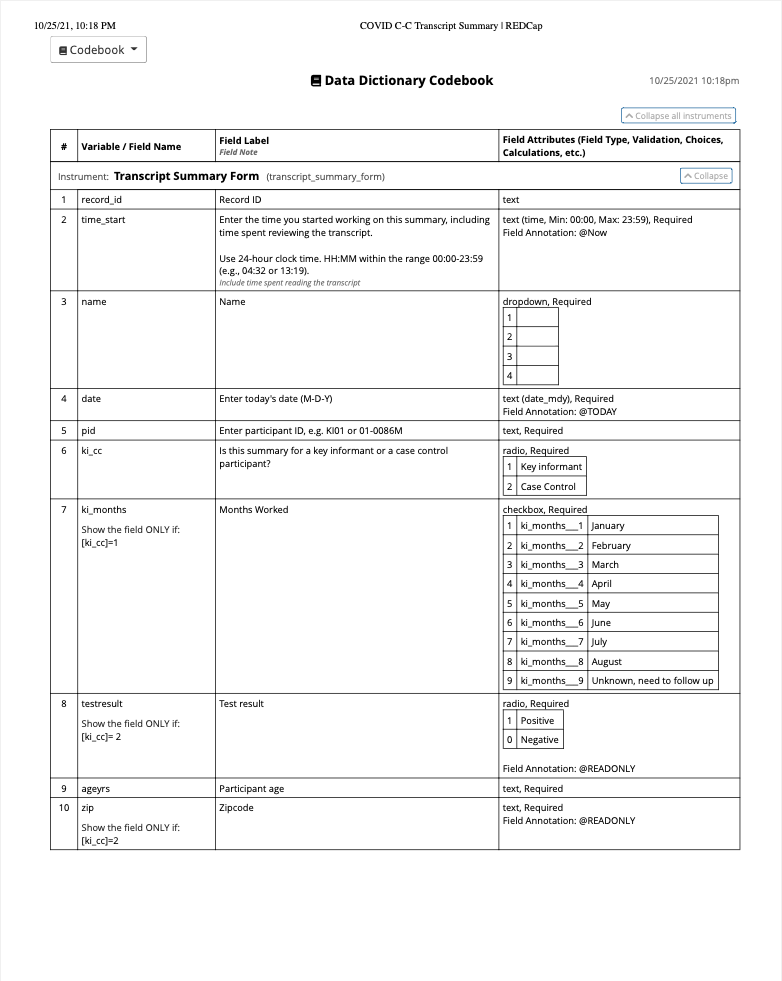


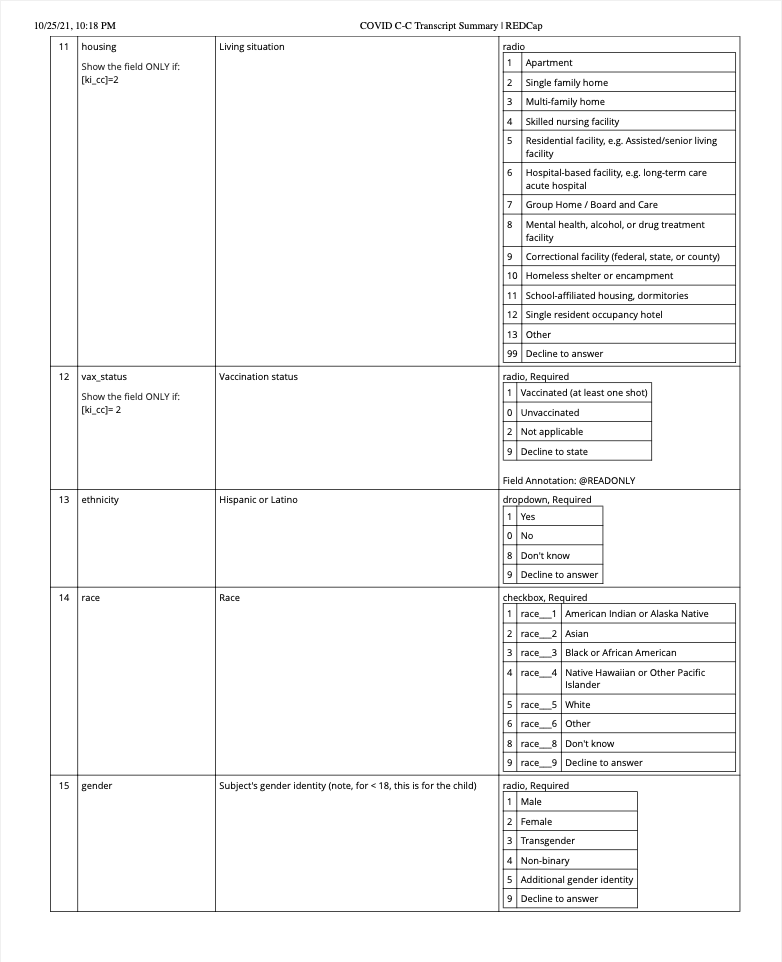


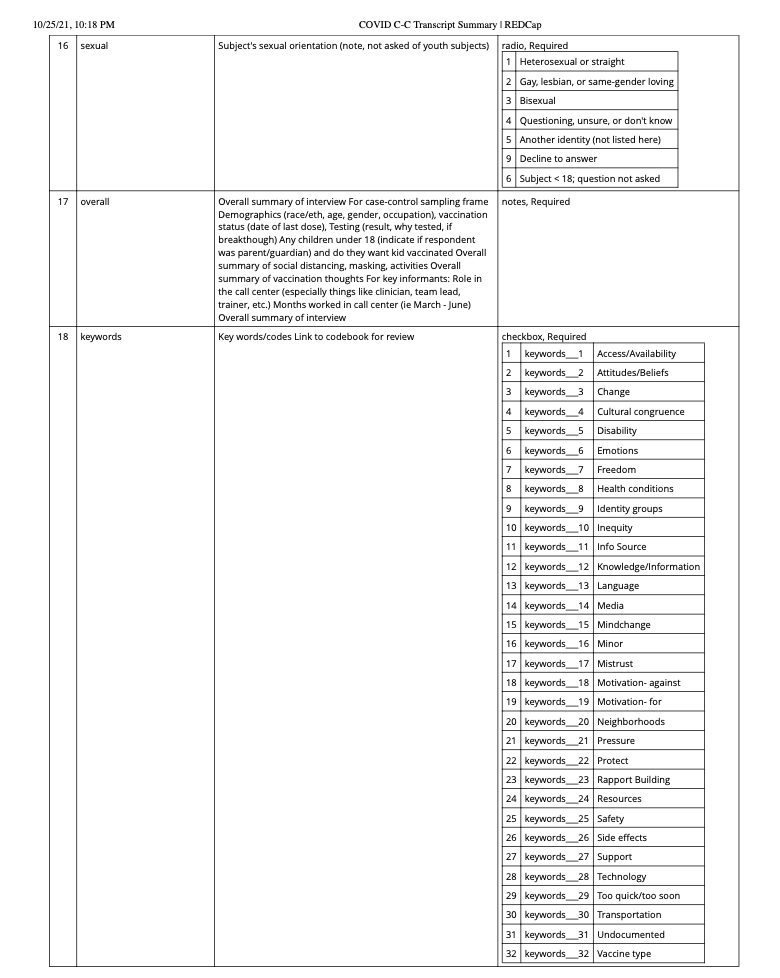


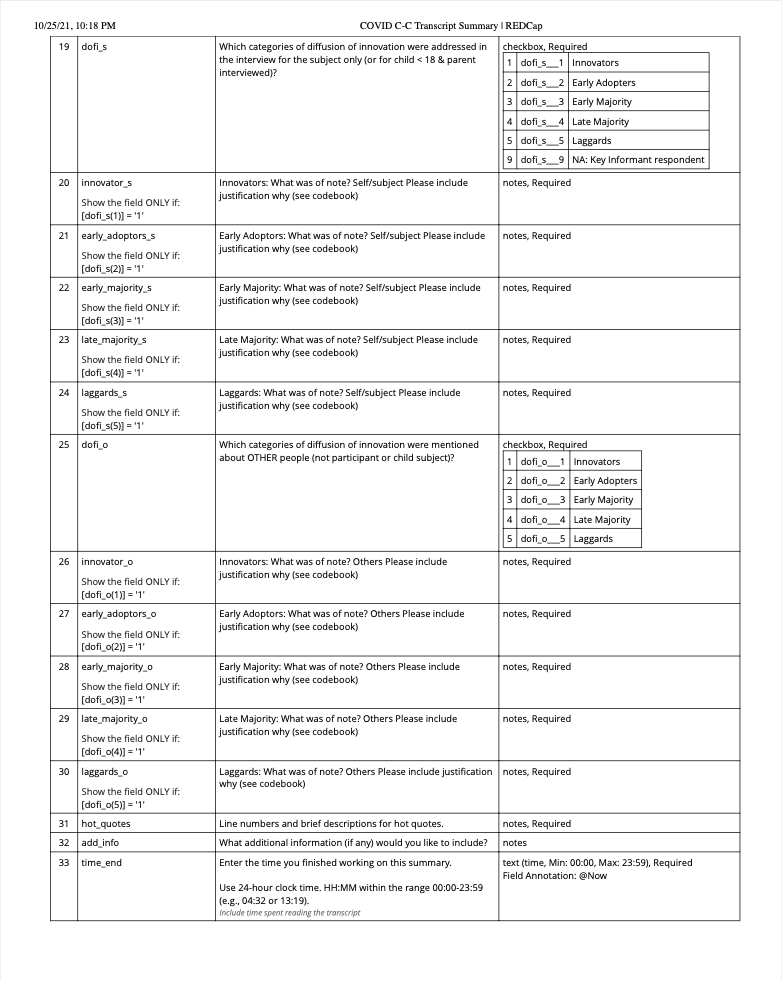


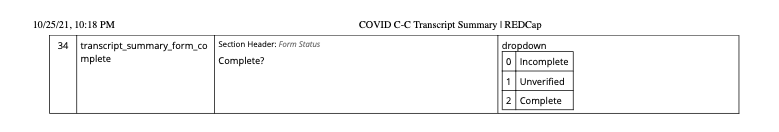

Supplement: sj-docx-2-inq-10.1177_00469580231159742 – Supplemental material for Factors Influencing Parent and Guardian Decisions on Vaccinating Their Children Against SARS-CoV-2: A Qualitative Study [file sj-docx-2-inq-10.1177_00469580231159742.docx]
